# Genome Assembly and Analysis of the North American Mountain Goat (*Oreamnos americanus*) Reveals Species-Level Responses to Extreme Environments

**DOI:** 10.1534/g3.119.400747

**Published:** 2019-12-05

**Authors:** Daria Martchenko, Rayan Chikhi, Aaron B. A. Shafer

**Affiliations:** * Environmental and Life Sciences Graduate Program, Trent University, K9J 7B8 Peterborough, Canada,; † Institut Pasteur and CNRS, C3BI USR 3756, Paris, France, and; ‡ Forensics Program, Trent University, K9J 7B8 Peterborough, Canada

**Keywords:** *de novo* genome assembly, HiRise scaffolding, demography, PSMC, mountain goat, Caprinae

## Abstract

The North American mountain goat (*Oreamnos americanus*) is an iconic alpine species that faces stressors from climate change, industrial development, and recreational activities. This species’ phylogenetic position within the Caprinae lineage has not been resolved and their phylogeographic history is dynamic and controversial. Genomic data could be used to address these questions and provide valuable insights to conservation and management initiatives. We sequenced short-read genomic libraries constructed from a DNA sample of a 2.5-year-old female mountain goat at 80X coverage. We improved the short-read assembly by generating Chicago library data and scaffolding using the HiRise approach. The final assembly was 2,506 Mbp in length with an N50 of 66.6 Mbp, which is within the length range and in the upper quartile for N50 published ungulate genome assemblies. Comparative analysis identified 84 gene families unique to the mountain goat. The species demographic history in terms of effective population size generally mirrored climatic trends over the past one hundred thousand years and showed a sharp decline during the last glacial maximum. This genome assembly will provide a reference basis for future population and comparative genomic analyses.

Mountain goats (*Oreamnos americanus*) are a symbol of alpine wilderness and belong to the Caprinae subfamily of ungulates (hoofed mammals) that are known for their exceptional climbing ability (Figure 1). As northern alpine specialists, mountain goats are vulnerable to climate change (Pettorelli *et al.* 2007) and face pressures from industrial development, recreational activities, and hunting (Côté and Festa-Bianchet 2003). There is a need to provide novel tools and information to support conservation and management initiatives as it pertains to this enigmatic species.

**Figure 1:**
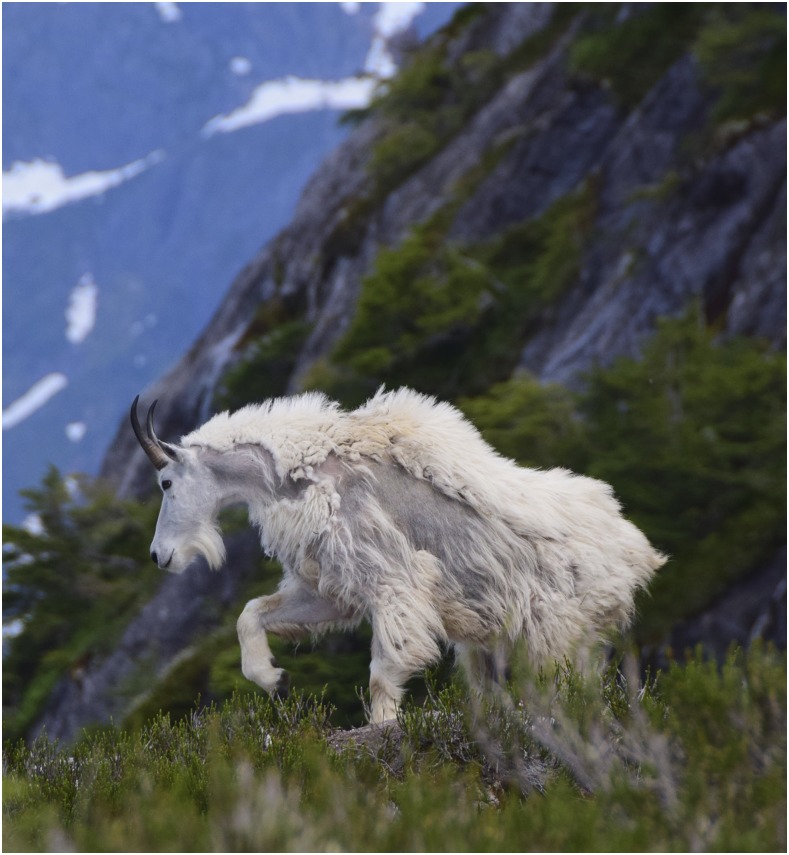
North American mountain goat female stomping to assert her dominance at Eulachon Creek, Alaska, USA. Mountain goats display a strong social structure and one of the highest levels of aggressiveness among ungulates. Photo by K.S. White.

Mountain goats are not true goats (*i.e.*, genus *Capra*), but diverged early within the Caprinae lineage and are typically associated with the chamois (*Rupicapra rupicapra*) and goral (*Naemorhedus* spp.); their phylogenetic position within the Caprinae is not resolved, but sampling of nuclear and mitochondrial genes suggested an early and independent divergence within the lineage (Shafer and Hall 2010). Similarly, the mountain goats phylogeographic history appears dynamic and is contentious (Shafer *et al.* 2011a, 2011b). Throughout the last glacial period most of the present-day range was covered with ice (Shafer *et al.* 2010), indicative of multiple refugia and contraction and expansion events of subpopulations (Shafer *et al.* 2011a). Within their present-day range, mountain goat populations are not continuous and dispersal is limited (Côté and Festa-Bianchet 2003), that combined with regional variability in habitat and climate, support the hypothesis of locally adapted populations across the range.

With the rapid development and lowering costs of sequencing technologies, the use of genomics in conservation and wildlife management is becoming more widespread. Over 20 ungulate genomes have been assembled (Martchenko *et al.* 2018), with chromosome-level assemblies for several commercially important ungulate species (ARS-UCD1.2, Oar_v3.1, ARS1), and an increasing number of draft assemblies for wild ungulates (Li *et al.* 2017; Zhang *et al.* 2018; Bana *et al.* 2018; Wang *et al.* 2019). This draft genome of the North American mountain goat adds to this list of ungulate genomes, and will be used for demographic analyses (*e.g.*, Miller *et al.* 2015), comparative genomic studies (*e.g.*, Orlando *et al.* 2013; Agaba *et al.* 2016), and testing for putative local adaptation (*e.g.*, Savolainen *et al.* 2013; Fitzpatrick and Keller 2015).

## Materials and Methods

### Library construction

A tissue sample was obtained from a spleen of a 2.5-year-old female harvested on Revillagigedo Island, Alaska, USA and frozen promptly after harvest. High quality molecular DNA was extracted using the phenol chlorophorm method (Sambrook and Russell 2006). Two TruSeq Nano genomic library preparations with an insert size of 500 bp were constructed and sequenced on an Illumina HiSeqX platform with 150 bp paired-end reads. A total of 214 Gbp of raw short-read data were generated.

We trimmed the adapters and the low quality bases from the reads with BBDuk (part of BBTools) (Bushnell 2018) to a minimum base quality 20 and a minimum read length 50 bp after a quality assessment with FastQC (Andrews 2010). To avoid any potential contamination of the genome with bacterial or viral sequences, we screened the trimmed reads with Kraken (Wood and Salzberg 2014) using the full standard database (Oct. 2017 release). A total of 0.95% of the reads were classified as belonging to a known bacterial or viral taxon and were removed. The final short-read dataset comprised 176 Gbp.

Three Chicago libraries were prepared (Dovetail Genomics) following the approach in Putnam *et al.* (2016). Briefly, Chicago library preparation involves *in vitro* chromatin reconstitution to generate libraries with separations between reads up to maximum fragment size of the input DNA. These libraries were sequenced on one lane of the Illumina HiSeqX instrument generating a total of 104 Gbp of sequence data.

### Genome assembly

A k-mer size of 101 was selected for the short-read dataset with KmerGenie (Chikhi and Medvedev 2014). The draft *de novo* assembly was completed with the Meraculous assembler v. 2.0.4 (Chapman *et al.* 2011) using the following options: ‘diploid_mode 1’, ‘min_depth_cutoff 0’ to allow for auto-detection of k-mer frequency cutoff by the assembler, ‘no_read_validation 0’ to decrease the runtime as the reads were trimmed and screened prior to the assembly, and ‘gap_close_aggressive 1’ to remove the uniqueness requirement and use the most common sequence obtained from potential gap-closing reads. For comparison purposes, the short-read data were also assembled with MEGAHIT v. 1.1.1 (Li *et al.* 2015) using ‘k-list 101’ option.

We improved the short-read assembly by scaffolding with the Chicago library data using the HiRise pipeline software (Putnam *et al.* 2016). The HiRise software uses a likelihood model of the Chicago data to infer the genomic distance between the read pairs, which is then used to scaffold and check the orientation of draft scaffolds.

### Quality assessment

To assess the assembly quality, we mapped the raw short-insert library reads to the assembled genome with bowtie2 (Langmead and Salzberg 2012). We also aligned the genome against itself with minimap2 (Li 2018) with options ‘-ax asm5 -X’ to check for putatively artifactual duplications, reflected by sequence overlaps between scaffolds (excluding self-hits). We ordered the SAM file by decreasing alignment scores in order to identify large regions that are similar between different scaffolds.

To validate the genome assembly and assess potential contamination and sequencing bias we analyzed the contig and scaffold assemblies with KAT (Mapleson *et al.* 2017). KAT plots the k-mer spectra – the number of distinct k-mers seen once, twice, three times etc. and compares it between the raw reads and the genome assembly. We calculated an assembly quality value (QV) following the method of Bickhart *et al.* (2017) and ran FRC_align (Vezzi *et al.* 2012) to identify any problematic regions in the genome. We also evaluated the quality of the assembled genome using BUSCO V3 and the mammalia odb9 database.

### Genome annotation

We identified and classified the repeat regions of the assembled genome using RepeatMasker v. 4.0.8 (Smit *et al.* 2013). We configured RepeatMasker with RMBlast v. 2.6.0 sequence search engine, Tandem Repeat Finder v. 4.0.9 (Benson 1999), Dfam_Consensus database (20181026 release) and RepBase RepeatMasker edition (20181026 release) (Bao *et al.* 2015) and used ‘-species artiodactyl’ parameter for the analysis.

We then annotated scaffolds that were greater than 2500 bp (n = 482) using both homology-based and *de novo* predictions. Proteins from *Homo sapiens*, *Equus caballus*, *Bos taurus*, and *Ovis aries* (all Ensembl 89 release (Hunt *et al.* 2018)) were aligned to genome using blastx v. 2.7.1. We also used NCBI mammalian RefSeq transcipt database v. 1.0 and BLAT v. 1.0 to help identify exon structure. For *de novo* prediction we first mapped RNAseq data (SRX1947618) from *Capra hircus*, the most closely related species available on NCBI Sequence Read Archive, to the genome using HISAT2 v. 2.10 (Kim *et al.* 2015) and used these data for prediction with Augustus v. 3.1.1 (Stanke *et al.* 2006). EVidenceModeler v. 1.1.1 (Haas *et al.* 2008) was used to generate a consensus gene set with the following default weighting scheme (Humann *et al.* 2018): gene prediction via Augustus (1x weight); protein alignments via blastx (5x weight); transcript alignment via blat (10x weight). Lastly, we used PASA v. 2.3.3 (Haas *et al.* 2008) to refine our gene identifications.

To assign function to the newly annotated genes we aligned them to the NCBI mammalian RefSeq protein database using blastp v. 2.7.1 with a maximum HSP distance of 30,000 and e-value of 1e^-8^. The Interpro v 5.29-68 and KEGG databases were screened to annotate domains and identify pathways based on the top blast hit. We used Infernal v. 1.1.1 (Nawrocki and Eddy 2013) and the Rfam database release 14.1 to annotate miRNA, rRNA, and snRNA genes; tRNAs were detected using tRNAscan-SE version 2.0.1 (Lowe and Eddy 1997).

### Species-specific genes, phylogeny and demographic history

Using the predicted protein sequences of *Oreamnos americanus*, we analyzed the orthologs shared between *Capra hircus*, *Equus caballus*, *Bos taurus*, *Sus scrofa*, *Ovis aries* and *Oreamnos americanus* with OrthoVenn (Wang *et al.* 2015). We also used PHYLUCE v. 1.5.0 (Faircloth 2016) to identify orthologous UCE sequences between *Sus scrofa*, *Equus caballus*, *Bos taurus*, *Capra hircus*, and *Ovis aries* and *Oreamnos americanus* using the 5k amniote probe set (Faircloth *et al.* 2012). We extracted UCEs with 250 bp flanks totaling 11.9 aligned Mbp. We conducted a maximum likelihood phylogenetic analysis using RAxML v. 8 (Stamatakis 2014) under a GTRGAMMAI substitution model selected with jModelTest 2 (Darriba *et al.* 2012). The phylogenetic tree was constructed with PAML MCMCtree v. 4.9 (Yang 2007) and calibrated with the divergence time of goat and cow (18.3–28.5 Mya) (Benton and Donoghue 2007).

Lastly, using a random 30X subset of the sequencing data, we modeled the historical effective population size (*N*_e_) for the North American mountain goat using PSMC [18]. We used the default parameters of 64 atomic time intervals (-p “4+25*2+4+6”), a generation time of 6 years (Li and Durbin 2011) and mutation rate µ= 1.33*10^−8^ mutations/site/generation, calculated as the average mammalian mutation rate of 2.22*10^−9^ mutations/site/year (Kumar and Subramanian 2002) multiplied by the generation time of 6 years.

### Data availability

The raw sequence data have been deposited in the Short Read Archive (SRA) under accession number PRJNA510081. This Whole Genome Shotgun project has been deposited at DDBJ/ENA/GenBank under the accession WJNR00000000. The version described in this paper is version WJNR01000000. Supplemental material available at figshare: https://doi.org/10.25387/g3.9884003.

## Results and Discussion

### Genome assembly

The short-read assembly with Meraculous (Chapman *et al.* 2011) produced a genome consisting of 172,540 scaffolds with an N50 of 29.0 Kb and total genome size of 2.5 Gbp. For comparison purposes, the short-read data were also assembled with MEGAHIT v. 1.1.1 (Li *et al.* 2015), which produced a less continuous contig assembly of 2.6 Gbp compared to the Meraculous contig assembly (Tables S1 and S2).

The final assembly including Chicago libraries was 2.5 Gbp in length and consisted of 3,217 scaffolds that have an N50 of 66.6 Mbp (Table 1). The assembled genome is 93% of the predicted size estimated by KmerGenie (Chikhi and Medvedev 2014). Compared to the other ungulate species, the N50 of the mountain goat assembly is in the top quartile and the assembly length is mid-range (Table S3) (Miller *et al.* 2015; Bickhart *et al.* 2017; Williams *et al.* 2017; Bana *et al.* 2018). The genomic GC content is 41.67%, compared to 41.97% for non-primate mammalian animals (Li and Du 2014).

**Table 1:** Genome assembly statistics for the North American mountain goat for the short-read data and subsequent scaffolding

	Short-read assembly	Scaffolded + Chicago assembly
Total length	2,489 Mbp	2,506 Mbp
N50 / L50	29.0 Kbp / 24,137 scaffolds	66,617 Kbp / 13 scaffolds
N90 / L90	6.5 Kbp / 92,814 scaffolds	18,734 Kbp / 37 scaffolds
Number of scaffolds	172,540	3,217
N count (% of genome)	530,049 (0.02%)	17,462,549 (0.70%)
Gaps	23,758	193,083

### Quality assessment

Mapping the sequencing reads back to the assembled genome can be used to identify misassemblies and check assembly quality (Muller *et al.* 2013). The overall alignment rate of mapped the raw short-insert library reads to the final scaffolded assembly was 97%; 80% of the reads aligned concordantly exactly 1 time and 14% of the reads aligned concordantly more than once (Table S4), indicating the high quality of the assembly. For reference, when the same analysis was completed for the high quality assembly of the domestic goat, 89% of the raw sequencing reads mapped back to the assembled genome (Dong *et al.* 2013). To detect potential long duplicated regions in the final scaffolded assembly, we aligned the genome assembly against itself. Sixteen out of 30,472 entries in the SAM file had scores over 1000, ranging from 1011 to 1488. By examining the CIGAR strings of each of those entries, we concluded that the assembly did not contain duplicated segments across scaffolds longer than 2 Kbp. The QV score of the genome was 41.8 and the FRC output was consistent with short-read domestic goat genome assemblies (Bickhart *et al.* 2017; Table S6).

For both contig and scaffold assemblies for the mountain goat (Fig. S1 and S2) the number of distinct k-mers (k = 27) with over 1X coverage, *i.e.*, k-mers that are not single-copy in the assembly, is low, which suggests low levels of sequencing bias and contamination. The 1X k-mer distribution has a much larger first peak, suggestive of an inbred individual, which is ideal for assembly purposes and why our sample was selected. This mountain goat is descended from a founding population of less than 17 individuals (Smith and Nichols 1984); further the mating system of mountain goats is such that only a small number of males sire most offspring (Mainguy *et al.* 2009), suggesting this individual should have low levels of heterozygosity.

A total of 4,104 single-copy orthologs were screened against the assembled mountain goat genome with 3,817 (93%) being complete, and 287 missing or fragmented BUSCOS (7%); this is in the same range as the BUSCO scores reported for other ungulate species (Li *et al.* 2017; Zhang *et al.* 2018). Similarly, we detected 471 of 481 ultraconserved elements (UCEs) (Bejerano *et al.* 2004) using scripts available in PHYLUCE v. 1.5.0 (Faircloth 2016) which is comparable to high quality mammalian genomes (Seemann *et al.* 2016).

### Genome annotation

Long interspersed nuclear elements (LINEs) comprised 27.2% of the assembled genome, short interspersed nuclear elements (SINEs) comprised 11.4%, and in total repeats represented 46.7% of the assembly (Table 2). The repeat content is consistent with other ungulate genomes (Dong *et al.* 2013; Li *et al.* 2017; Zhang *et al.* 2018).

**Table 2:** Summary of repeats in the assembled genome of *Oreamnos americanus*

	Length (bp)	Percentage of sequence
Total interspersed repeats:	1,146,349,974	45.74%
SINEs	286,943,974	11.45%
LINEs	681,473,093	27.19%
LTR elements	122,655,871	4.89%
DNA elements	54,579,895	2.18%
Unclassified	697,141	0.03%
Satellites	3,429,492	0.14%
Simple repeats	17,168,694	0.69%
Low complexity	3,546,216	0.14%
Total:	1,170,891,890	46.72%

Our annotation pipeline of the longest scaffolds resulted in 22,292 total genes with 16,012 being protein coding. Of these, the Interpro v 5.29-68 databases identified 13,874 (74%) genes with information, and 9,460 (51%) having a gene ontology assignment. Similarly, RefSeq protein database detected a a total of 14,470 genes (78%) with NCBI RefSeq match. Lastly, the Interpro v 5.29-68 databases annotated 13,874 (74%) genes with information, with 9,460 (51%) having a gene ontology assignment. A total of 34,819 putative noncoding RNA sequences were identified encompassing 0.1% of the genome and is comparable to other ungulates (Zhang *et al.* 2018).

### Species-specific genes, phylogeny and demographic history

**Figure 2:**
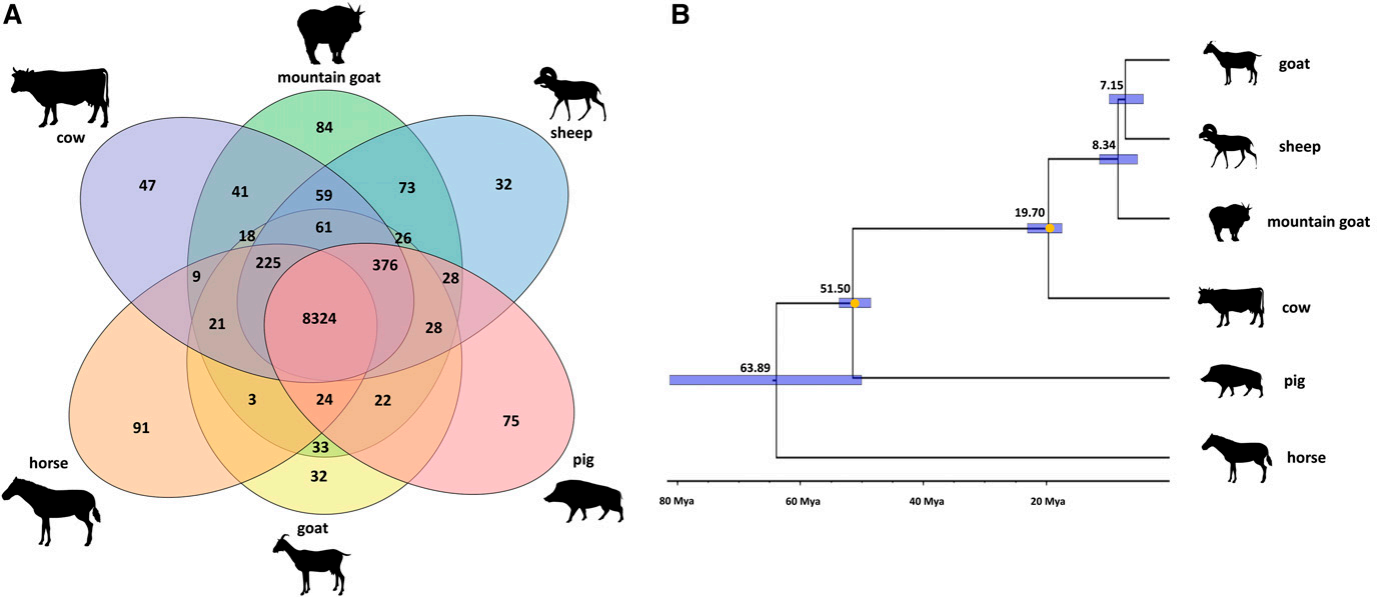
a) A Venn diagram of orthologous clusters shared among mountain goat (*Oreamnos americanus)*, pig (*Sus scrofa)*, horse *(Equus caballus)*, cow *(Bos taurus)*, goat *(Capra hircus)* and sheep (*Ovis aries)*. The numbers represent the number of orthologous clusters and only the clusters unique to each species and shared with *O. americanus* are labeled for clarity. b) Phylogenetic relationships and divergence times between the six species estimated from ultraconserved elements (UCEs) and MCMCtree. The calibration points are indicated by orange dots; node bars encompass the 95% credible intervals.

A total of 84 gene families were found to be specific to the mountain goat (Figure 2a). Seventy-three orthologous clusters were uniquely shared between *Ovis aries* and *O. americanus*, 33 between *Capra hircus* and *O. americanus*, and 41 between *Bos taurus* and *O. americanus*. The gene families unique to mountain goat were enriched for ferroxidase activity, transcription regulation and protein folding and stability; we hypothesize that enrichment for the ferroxidase activity could potentially have allowed mountain goats to adapt to their alpine environment due to its link to erythropoiesis and altitude (Cherukuri *et al.* 2004).

**Figure 3:**
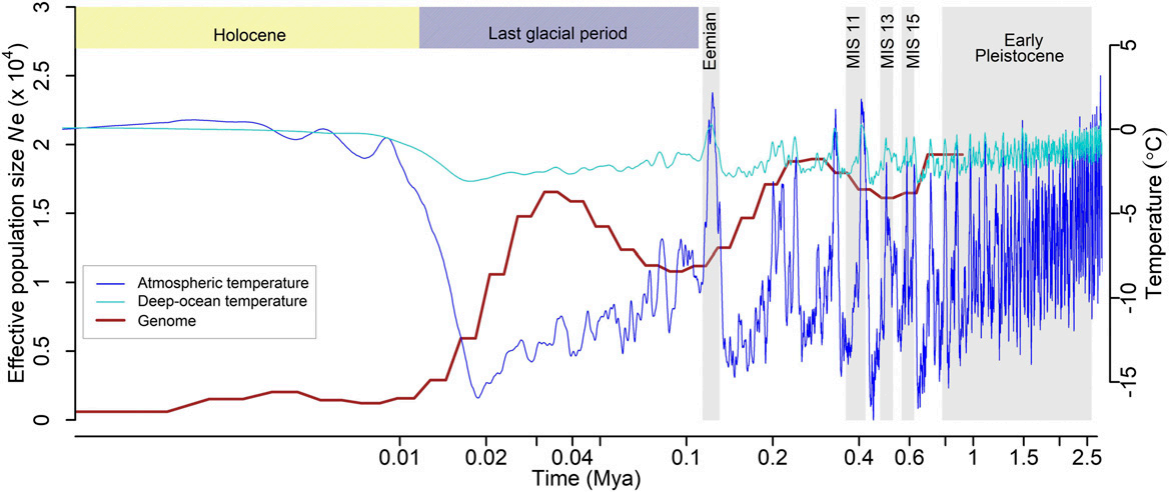
Historical effective population size (*N*_e_) of the North American mountain goat estimated with PSMC analysis assuming a mutation rate *µ* of 1.33*10^−8^ mutations/site/generation and a generation time of 6 years. Atmospheric and ocean temperatures in °C are given on the vertical right axis, *N*_e_ in units of 10,000 individuals on the left vertical axis, and time in Mya on the logarithmic horizontal axis.

The phylogenetic analysis (Figure 2b) supports the mountain goat lineage as ancestral to sheep and goats as expected (Shafer and Hall 2010). There was no relationship between the assembly strategy, number of indels in UCEs, and phylogenetic placement (Table S7). The mitochondrial genome phylogeny confirmed a similar relationship (Fig. S3). As the ice sheets progressed during the last glacial period, the Earth experienced a decrease in average surface temperature; the mountain goat *N*_e_ reflects these temperature patterns, with a general downward trend in *N*_e_ from 200 kya to 35 kya (Figure 3). PSMC analysis suggested a near 10-fold decrease in the *N*_e_ of mountain goats at the end of the last glacial period and the start of the Holocene likely due to warming and loss of suitable habitat.

### Conclusion

This is the first genome assembly and annotation for the North American mountain goat, and the first *de novo* assembly of a wild caprid. The biological sample came from an island with a small founding population (Smith and Nichols 1984) making it ideal for genome assembly. Using the newly assembled genome we identified species-specific genes and modeled the historical population demography of the species that showed a dramatic decline at the height of the last glaciation. Relative to other ungulate genomes, including economically valuable domesticated species that incorporated long-read sequencing strategies, this is among the highest quality wild ungulate genomes to date (Martchenko *et al.* 2018).
